# Identification and Functional Analysis of Candidate Genes Influencing Citrus Leaf Size Through Transcriptome and Coexpression Network Approaches

**DOI:** 10.3390/genes16010097

**Published:** 2025-01-17

**Authors:** Xiaoxiao Wu, Yuanhui Xiao, Ping Liu, Qiuling Pang, Chongling Deng, Cuina Fu, Haimeng Fang, Chuanwu Chen

**Affiliations:** Guangxi Key Laboratory of Germplasm Innovation and Utilization of Specialty Commercial Crops in North Guangxi, Guangxi Citrus Breeding and Cultivation Technology Innovation Center, Guangxi Academy of Specialty Crops, Guilin 541004, China; 15578386736@126.com (X.W.); xiaoyh1283@163.com (Y.X.); liupingsmile@126.com (P.L.); 13193698720@163.com (Q.P.); cldeng88168@126.com (C.D.); fucuina99@163.com (C.F.); fanghaimeng1029@gmail.com (H.F.)

**Keywords:** citrus, leaf size, transcriptome sequencing, WGCNA, candidate genes

## Abstract

Background: Leaves are the main organs involved in photosynthesis. They capture light energy and promote gas exchange, and their size and shape affect yield. Identifying the regulatory networks and key genes that control citrus leaf size is essential for increasing citrus crop yield. Methods: In this study, transcriptome sequencing was performed on three leaf materials: the ‘Cuimi’ kumquat (Nor) variety and its leaf variants, larger-leaf (VarB) and smaller-leaf (VarS) varieties. Results: Correlation and principal component analyses revealed a relatively close correlation between Nor and VarS. A total of 7264 differentially expressed genes (DEGs), including 2374 transcription factors (TFs), were identified, and 254 DEGs were common among the three materials. GO and KEGG enrichment analyses revealed significant enrichment in glucose metabolism, cell wall composition, starch biosynthesis, and photosynthesis pathways. WGCNA identified three specific modules related to the different leaf sizes of these three citrus materials. Fifteen candidate genes related to leaf size, including three transcription factors, *Fh5g30470* (MYB), *Fh7g07360* (AP2/ERF), and *Fh5g02470* (SAP), were identified on the basis of connectivity and functional annotations. Conclusions: These findings provide a theoretical foundation for a deeper understanding of the molecular mechanisms underlying citrus leaf size and offer new genetic resources for the study of citrus leaf size.

## 1. Introduction

Citrus holds a prominent economic position in China as the fruit with the largest cultivation area and yield [1]. Leaves play a pivotal role in photosynthesis and are essential plant organs that show significant morphological diversity, originating from the shoot apical meristem (SAM) [2]. Leaf size directly impacts light capture and photosynthesis levels [3]. Larger leaves can absorb more light energy, can accommodate more chlorophyll, and have a larger surface area, thereby improving the plant’s utilization of light and increasing the efficiency of photosynthesis and thereby obtaining more nutrients and energy to support growth [4]. In general, leaves are essential to the growth and survival of plants, so changes in leaf size reflect natural selection for functional operation; thus, leaf size is considered closely related to leaf photosynthesis. Therefore, to maximize the capture of light energy, plants widen their leaves as much as possible, and to facilitate gas exchange, leaves need to be as thin and flat as possible [5]. However, leaves do not widen unconditionally; if the leaves are too large, they will be overexposed to the sun, resulting in excessive leaf temperature. Moreover, the leaf shape and vein pattern are closely related, influencing each other during their development. Thus, leaf development and the size of the leaf area are key factors influencing crop growth and development, stress resistance, and yield. These factors are vital for research on physiology, biochemistry, genetic breeding, and crop cultivation and serve as indicators of crop growth, development, yield formation, and variety characteristics [6]. The regulatory mechanisms governing leaf size have been widely investigated, with a focus on four key cell development processes: cell quantity in the leaf primordium, the cell proliferation rate and duration, the cell expansion rate and duration, and the extent of meristem division [7,8,9]. Variations in these processes can impact either cell quantity or cell size, ultimately influencing the overall size of leaves. Precise regulation of cell proliferation and cell expansion is essential for determining leaf size. However, the molecular mechanisms influencing citrus leaf size remain unclear, as this trait is complex and is affected by the expression of multiple genes and environmental factors.

Advancements in sequencing technology have provided increasing evidence that RNA transcriptional regulation plays an important role in plant growth and development [10,11]. RNA-seq, a popular second-generation high-throughput sequencing technique, is extensively used in plant growth and development research [10,11,12]. Coexpression network analysis, a systems biology approach, analyzes gene expression correlations and constructs a gene coexpression network to identify functionally relevant gene modules [12,13,14]. For example, the gene regulatory network of banana fruit ripening transcriptome analysis has been revealed [15]. Additionally, a transcriptional regulatory network was constructed using a chickpea time series transcriptome dataset, identifying key modules and candidate genes for seed size/weight [16]. RNA-seq of citrus from six developmental stages (the last of which was the mature fruit stage) and four major tissues (yellow cortex, white cortex, tunicate, and juice cells) revealed the role of the abscisic acid (ABA) signaling regulatory network during citrus fruit development and ripening. Several key genes involved in sucrose accumulation and citric acid metabolism have been identified [17]. Through a pangenome map of the citrus subfamily combined with transcriptome analysis, the transcription factor *CitPH4*, which contains an R2R3-MYB domain, was identified. *CitPH4* promotes citric acid accumulation by binding to and activating the tonoplast proton pump gene *PH5* [18]. WGCNA of transcriptome data from ‘Newhall’ oranges revealed that the transcription factor *CsESE3* was coexpressed with multiple lipid metabolism pathway genes, suggesting its involvement in the synthesis of citrus waxes and jasmonic acid (JA) [19].

Research on citrus plants has focused predominantly on stress resistance and fruit development, whereas few studies have addressed leaf-related regulatory mechanisms and related gene functions [20,21,22]. Leaves, as crucial photosynthetic organs, are vital for plant growth and are the most important “source” of photosynthesis. Previous studies have explored the correlation between leaf size and morphology in model plants such as rice and *Arabidopsis thaliana*, but no reports have addressed this correlation in citrus [23,24,25]. To fill this gap, RNA-seq sequencing of leaves from ‘Cuimi’ kumquat (Fh, *Fortunella hindsii*) (Nor) and its leaf variants—larger-leaf (VarB) and smaller-leaf (VarS)—was conducted. Cluster differential expression analysis was performed on the sequencing data. In this study, a weighted gene coexpression network was constructed, and potential candidate genes associated with citrus leaf size were identified. This research offers a theoretical basis for advancing our comprehension of the molecular mechanisms controlling citrus leaf size and provides new genetic resources for citrus research.

## 2. Materials and Methods

### 2.1. Plant Material

The experimental materials selected in this study were Nor ‘Cuimi’ kumquats (variety ‘Cui Mi Jin Gan’ produced by a natural variation in the Huapi Jin Gan in Rong’an County, approval number: Guishen Guo 2014003) and three materials with enlarged leaves (VarB) and smaller leaves (VarS). Leaves were collected from each material (each sample was replicated 6 times, 3 replicates were used for RNA-seq, and 3 replicates were used for qRT-PCR), and the samples were quickly frozen in liquid nitrogen for subsequent experiments.

### 2.2. RNA Extraction, cDNA Library Preparation, and Sequencing

RNA was isolated using the TRIzol method, and its quality was assessed via 1% agarose gel electrophoresis. The total RNA was preserved at −80 °C and subsequently shipped on dry ice to Xinjiang Aidesen Biotechnology Co., Ltd. (Urumqi, China) for sequencing. A total of 1 μg of RNA per sample was used as input material for the RNA sample preparations. The sequencing libraries were generated via the Hieff NGS Ultima Dual-mode mRNA Library Prep Kit for Illumina (Yeasen Biotechnology Co., Ltd., Shanghai, China). mRNA was enriched using oligo (dT)-containing magnetic beads, which bind to the polyA tail of mRNA through A–T complementary pairing. Subsequently, a fragmentation buffer was introduced to cleave the mRNA into shorter fragments. The initial cDNA strand was generated via six-base random hexamers (Invitrogen, Carlsbad, CA, USA), with the mRNA serving as the template. A mixture of buffer, dNTPs, and DNA polymerase I was subsequently employed to synthesize the second cDNA strand. The resulting double-stranded cDNA was purified via AMPure XP beads (Beckman Coulter, Beverly, CA, USA). Then, 3 µL of USER Enzyme (NEB, Ipswich, MA, USA) was used with size-selected, adaptor-ligated cDNA at 37 °C for 15 min, followed by 5 min at 95 °C before PCR. Then, PCR was performed with Phusion High-Fidelity DNA polymerase, universal PCR primers and Index (X) primers. Finally, the PCR products were purified (AMPure XP system), and library quality was assessed on an Agilent Bioanalyzer 2100 system. Once the library’s effective concentration exceeded 2 nM, sequencing in PE150 mode was carried out using the Illumina HiSeq 2500 platform. The data were subjected to filtering and quality control procedures via fastp software (version 0.23.4), with the obtained clean data being employed for subsequent analysis [26]. The reads were aligned in HISAT2 using the citrus genome Hongkong kumquat (https://www.ncbi.nlm.nih.gov/datasets/genome/GCA_004802465.1/ (accessed on 15 November 2023)) as a reference, and String Tie was used to quantify the aligned reads [27,28].

### 2.3. Identification of Differentially Expressed Genes

Gene expression levels were quantified using the fragments per kilobase of transcript per million mapped reads (FPKM) method. DESeq2 was then applied to determine the differential expression of genes across various samples on the basis of their expression levels [29]. An FDR ≤ 0.01 and an absolute value of log2-fold change ≥ 1 were used as the standards for screening differentially expressed genes (DEGs) [30]. The amino acid sequences of all the DEGs were submitted to the KEGG (https://www.genome.jp/kegg/ (accessed on 28 January 2024)) database. The whole-genome sequence of citrus was submitted to PlantTFDB (http://planttfdb.cbi.pku.edu.cn/ (accessed on 7 February 2024)) for transcription factor prediction.

### 2.4. Construction of the Coexpression Network

The gene expression profiles (FPKM) of the differentially expressed genes (DEGs) were analyzed using the dynamic branch cutting method in the R language WGCNA package [31]. To achieve a scale-free network distribution, the weight coefficient β should exhibit a correlation coefficient close to 0.8 and possess a specific level of gene connectivity. In this study, a weight coefficient of β = 4 was chosen. The network was established via the automatic network construction function for blockwise modules. Various valid modules were generated, each containing a different number of genes. Modules exhibiting a similarity of 0.75 were merged using minModuleSize = 30 and Merge Cut Height = 0.25 as the criteria. The module eigengene (ME) characteristic vector and correlation coefficients between different samples were then computed. The coexpression network was visualized using Cytoscape (version 3.10) software. The gene interaction network of key modules was constructed using Cytoscape 3.10 software, and the MCC algorithm of the CytoHubba plug-in in the software was used to screen the top five hub genes in the network as candidate genes [32].

### 2.5. qRT-PCR

Total RNA was isolated via an RNA extraction kit (Tiangen, Beijing, China). The concentration of each RNA sample was assessed using a NanoDrop 2000 spectrophotometer (Thermo Fisher Scientific, Waltham, MA, USA), and the integrity of the RNA was verified via gel electrophoresis. One microgram of isolated RNA was subsequently used to obtain first-strand cDNA via reverse transcription using the PrimeScript™ RT Kit with gDNA Eraser (Takara Bio Inc., Shiga, Japan). qRT-PCR analysis was performed using a Roche LC480 instrument (Roche Diagnostics GmbH, Mannheim, Germany) and SYBR Green (Takara Bio Inc.). A two-step PCR amplification program was employed, including initial denaturation at 95 °C for 30 s, followed by 40 cycles of denaturation at 95 °C for 5 s and annealing at 60 °C for 35 s. Roche LC480 software automatically generated amplification, melting, and standard curve data. The results were analyzed for relative quantification using the 2^−ΔΔCt^ method [33]. The internal reference gene was *β-actin* [34] (GenBank: cb250364), and three biological replicates were performed for each program. All primers used in this study are listed in Table S1.

## 3. Results

### 3.1. Overall Analysis of RNA-Seq Data

**Figure 1:**
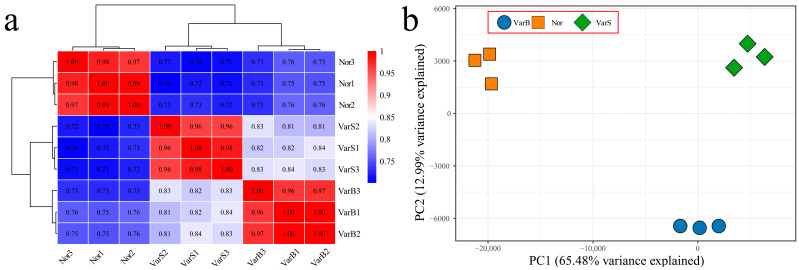
(**a**) Correlation and cluster analyses of nine RNA-seq samples from citrus; the redder the color is, the higher the correlation coefficient, and the bluer the color is, the lower the correlation coefficient. (**b**) Principal component analysis of nine RNA-seq samples from citrus; different colors represent different samples: orange represents Nor, blue represents VarB, and green represents VarS.

RNA-seq was performed on nine samples from three materials, yielding 121.73 Gb of raw bases. After filtering, a total of 117.33 Gb of effective bases were obtained. Each sample produced at least 8.96 Gb of effective bases. The Q20 base percentages ranged from 98.18% to 98.85%, with an average of 98.38%. The Q30 base percentages ranged from 94.89% to 96.80%, with an average of 95.51%. The alignment rates with the reference genome ranged from 92.67% to 95.86%, with an average alignment rate of 94.21% (Table S2). The correlation coefficients between the biological replicates exceeded 0.96 (Figure 1a), indicating high reproducibility. Cluster analysis placed Nor and VarS on the same branch, with correlation coefficients greater than 0.81. The results of principal component analysis (PCA) confirmed that the biological replicates clustered together, with Nor and VarS showing close relationships (Figure 1b). In summary, the RNA-seq data were reliable and reproducible, and the relationships between Nor and VarS were relatively close.

**Figure 2:**
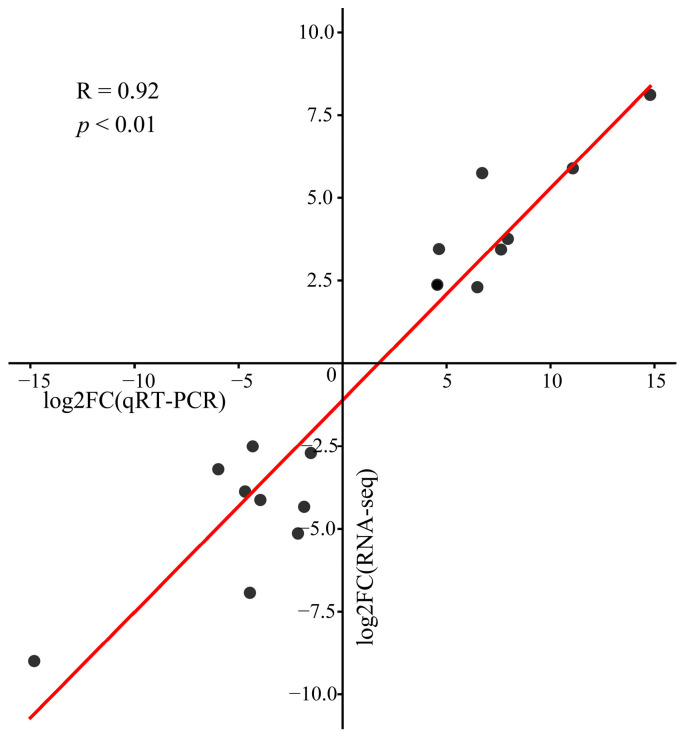
Scatter plot of the correlations of the transcriptome data with the qRT-PCR gene expression levels.

To confirm the accuracy of the transcriptome expression profiles, qRT-PCR validation of six randomly selected genes confirmed a significant correlation with the RNA-seq data (R = 0.92, *p* < 0.01), indicating the reliability of the transcriptome sequencing data (Figure 2).

### 3.2. Differential Expression Analysis

**Figure 3:**
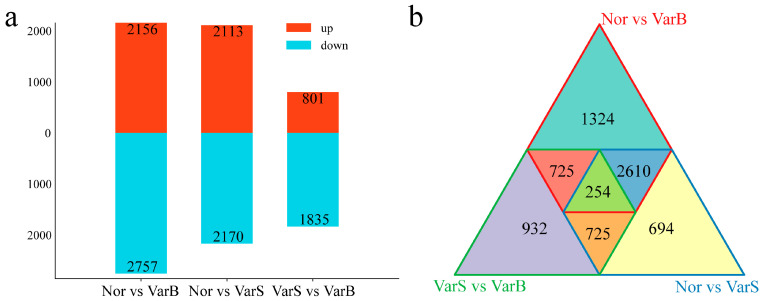
(**a**) Numbers of DEGs between different materials. (**b**) Venn diagram of the DEGs between different materials.

To investigate the transcriptional regulation of citrus leaf size, we identified differentially expressed genes (DEGs) in the leaves of different materials. There were 4913 differentially regulated genes identified between Nor and VarB, with 2156 upregulated and 2757 downregulated genes. Among the Nor and VarS genes, 4283 differentially regulated genes were identified, among which 2113 were upregulated and 2170 were downregulated. There were 2636 differentially regulated genes identified between VarS and VarB, among which 801 were upregulated and 1835 were downregulated (Figure 3a). Unique DEGs numbered 932 between VarS and VarB, 694 between Nor and VarS, and 1324 between Nor and VarB (Figure 3b). A total of 254 DEGs were common across all three materials.

### 3.3. Enrichment Analysis

**Figure 4:**
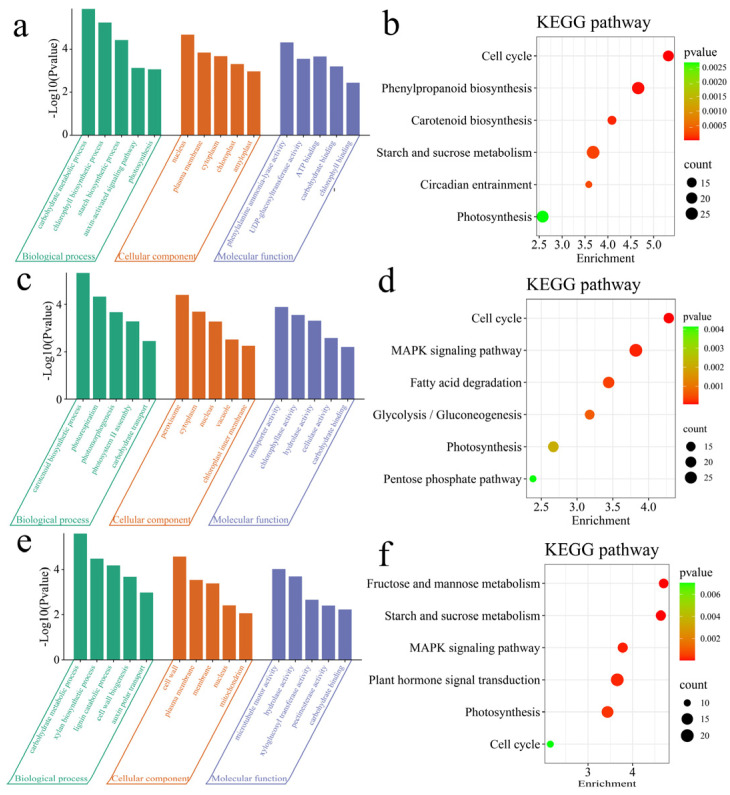
(**a**) GO enrichment analysis of DEGs between Nor and VarB. (**b**) KEGG enrichment analysis of DEGs between Nor and VarB. (**c**) GO enrichment analysis of DEGs between Nor and VarS. (**d**) KEGG enrichment analysis of DEGs between Nor and VarS. (**e**) GO enrichment analysis of DEGs between VarS and VarB. (**f**) KEGG enrichment analysis of DEGs between VarS and VarB.

To clarify the functions of the DEGs, GO and KEGG enrichment analyses were performed on the DEGs between materials (Figure 4). The 4913 DEGs between Nor and VarB were significantly enriched in the following biological processes: the carbohydrate metabolic process, chlorophyll biosynthetic process, starch biosynthetic process, the auxin-activated signaling pathway, and photosynthesis (Figure 4a). The 4913 DEGs between VarS and VarB were significantly annotated in the cell cycle, phenylpropanoid biosynthesis, carotenoid biosynthesis, starch and sucrose metabolism, circadian entrainment, and photosynthesis pathways (Figure 4b). The 4283 DEGs between Nor and VarS were significantly enriched in the following biological processes: carotenoid biosynthetic process, photorespiration, photomorphogenesis, photosystem II assembly, and carbohydrate transport (Figure 4c). The 4283 DEGs between Nor and VarS were significantly annotated in the cell cycle, the MAPK signaling pathway, fatty acid degradation, glycolysis/gluconeogenesis, the pentose phosphate pathway, and the photosynthesis pathway (Figure 4d). The 2636 DEGs between VarS and VarB were significantly enriched in the following biological processes: the carbohydrate metabolic process, xylan biosynthetic process, lignin catabolic process, cell wall biogenesis, and auxin polar transport (Figure 4e). The 2636 DEGs between VarS and VarB were significantly annotated in plant hormone signal transduction, starch and sucrose metabolism, the MAPK signaling pathway, fructose and mannose metabolism, and the cell cycle and the photosynthesis pathway (Figure 4f).

**Figure 5:**
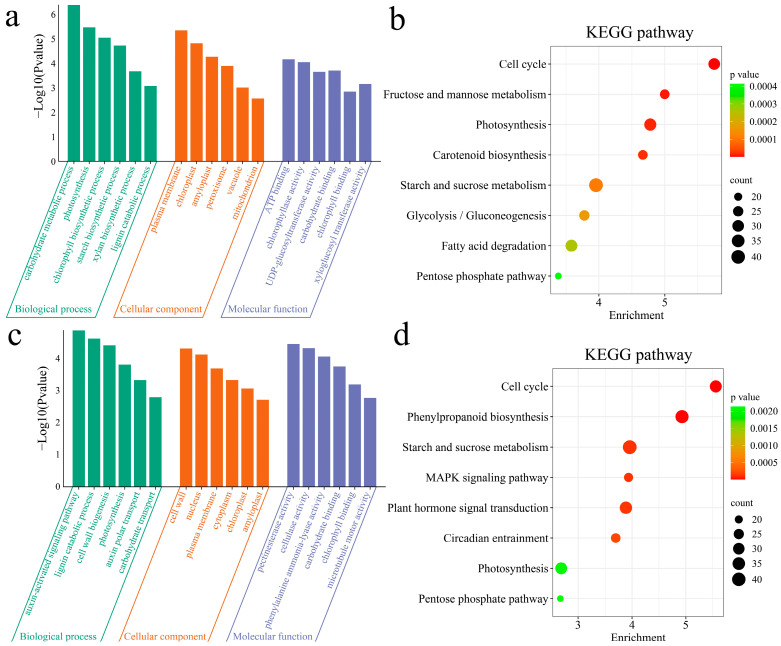
(**a**) GO enrichment analysis of upregulated DEGs. (**b**) KEGG enrichment analysis of upregulated DEGs. (**c**) GO enrichment analysis of downregulated DEGs. (**d**) KEGG enrichment analysis of downregulated DEGs.

GO and KEGG enrichment analyses were performed on the upregulated and downregulated DEGs (Figure 5). The biological processes in which the upregulated DEGs were significantly enriched were carbohydrate metabolic process, photosynthesis, chlorophyll biosynthetic process, starch biosynthetic process, xylan biosynthetic process, and lignin catabolic process (Figure 5a). The upregulated DEGs were significantly enriched in the cell cycle, carotenoid biosynthesis, starch and sucrose metabolism, photosynthesis, fructose and mannose metabolism, fatty acid degradation, glycolysis/gluconeogenesis, and the pentose phosphate pathway (Figure 5b). The biological processes in which the downregulated DEGs were significantly enriched were the auxin-activated signaling pathway, lignin catabolic process, cell wall biogenesis, photosynthesis, auxin polar transport, carbohydrate transport and the cell wall (Figure 5c). The upregulated DEGs were significantly enriched in the cell cycle, phenylpropanoid biosynthesis, starch and sucrose metabolism, circadian entrainment, photosynthesis, plant hormone signal transduction, the MAPK signaling pathway, and the pentose phosphate pathway (Figure 5d).

### 3.4. Transcription Factor Analysis

**Figure 6:**
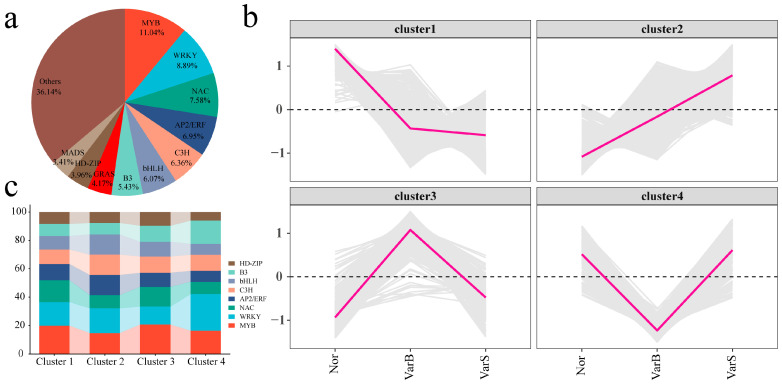
(**a**) Percentage pie chart of differentially expressed transcription factors. (**b**) Line plot of differentially expressed transcription factor expression patterns. (**c**) Percentages of TOP transcription factors in different clusters.

A total of 2374 transcription factors, including the MYB, WRKY, NAC, AP2/ERF, C3H, bHLH, B3, GRAS, HD-ZIP, and MADS families, were identified among the 7264 DEGs (Figure 6a). Hierarchical clustering divided the transcription factors into four distinct expression patterns (Figure 6b). Cluster 1 presented the lowest expression level in VarS and the highest expression level in Nor. Cluster 2 presented the lowest expression level in Nor and the highest expression level in VarS. Cluster 3 had the lowest expression levels in Nor and VarS and the highest expression level in VarB. Cluster 4 presented the lowest expression level in VarB and the highest expression levels in Nor and VarS. MYB and WRKY accounted for the greatest proportion of genes in Cluster 1 (19.96% and 16.63%, respectively) (Figure 6c). The highest proportions of WRKY and MYB in Cluster 2 were 17.49% and 14.75%, respectively. The highest proportions of MYB and NAC in Cluster 3 were 20.73% and 13.82%, respectively. The highest proportions of WRKY, B3, and MYB in Cluster 4 were 25.86%, 16.38%, and 16.38%, respectively.

### 3.5. Construction of a Weighted Gene Coexpression Network

**Figure 7:**
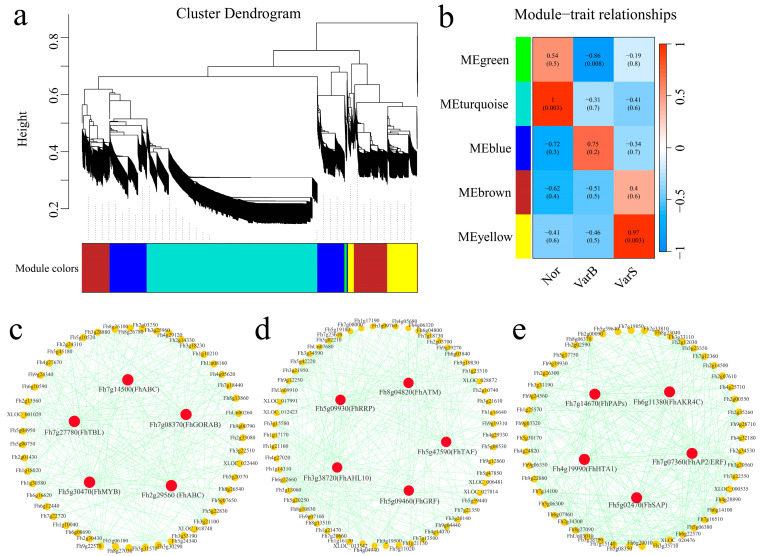
(**a**) Hierarchical clustering tree of genes identified via coexpression network analysis. (**b**) Heatmap of correlations and significance between modules and different samples. (**c**) Gene coexpression network within the green module. (**d**) Gene coexpression network within the turquoise module. (**e**) Gene coexpression network within the yellow module.

Weighted gene coexpression network analysis (WGCNA) identified 7264 DEGs across the materials, forming five coexpression modules using a β soft threshold of four (scale-free R^2^ > 0.8). The dynamic cutting tree method was used to merge modules with similar expression levels, and a total of five coexpression modules were obtained. The modules were color-coded, with the green module highly correlated with VarB (r < −0.8, *p* < 0.05), the turquoise module correlated with Nor (r > 0.8, *p* < 0.05), and the yellow module correlated with VarS (r > 0.8, *p* < 0.05) (Figure 7a,b). Gene interaction networks were constructed for the green, turquoise and yellow modules, identifying five hub genes per module. The hub genes included *Fh7g14500*, *Fh7g27780*, *Fh7g08370*, *Fh5g30470*, and *Fh2g29560* in the green module (Figure 7c); *Fh5g09930*, *Fh8g04820*, *Fh3g38720*, *Fh5g42590*, and *Fh5g09460* in the turquoise module (Figure 7d); and *Fh7g14670*, *Fh6g11380*, *Fh4g19990*, *Fh7g07360*, and *Fh5g02470* in the yellow module (Figure 7e).

To further explain the relationships between the 15 hub genes and citrus leaf size, the hub genes were aligned to the Arabidopsis genome (Genome version Araport11, https://www.arabidopsis.org/download/list?dir=Sequences%2FAraport11_blastsets (accessed on 3 March 2024)) using BLAST), and the functions of the hub genes were annotated on the basis of homologous Arabidopsis genes (Table 1). Functional annotation revealed key roles in the regulation of leaf size via the transcription factors *Fh5g30470* (MYB), *Fh7g07360* (AP2/ERF), and *Fh5g02470* (SAP). The Arabidopsis homologous gene AT1G01520 of *Fh5g30470* is involved in regulating circadian rhythm, the Arabidopsis homologous gene AT2G46310 of *Fh7g07360* is involved in regulating the development of cotyledons and leaves, and the Arabidopsis homologous gene AT5G35770 of *Fh5g02470* is involved in regulating the development of inflorescences, flowers, and ovules. *Fh7g14500* encodes an ATP-binding cassette transporter protein (ABC) and is involved in regulating growth and development. *Fh7g27780* encodes Trichome birefringence-like (TBL), which is involved in the synthesis and deposition of secondary wall cellulose. *Fh7g08370* encodes the microtubules required for immunity. *Fh2g29560* encodes an ATP-binding cassette (ABC), which mainly regulates growth and development. *Fh5g09930* encodes the rRNA biogenesis RRP-like protein (RRP), which is involved in rRNA processing and capping in the nucleus and cytoplasm. *Fh8g04820* encodes Arabidopsis thioredoxin M-type (ATM), which is involved in activating the cell cycle and DNA damage repair. *Fh3g38720* encodes AT-Hook-Like10 (AHL10), which is able to inhibit the maturation of the shoot apical meristem. *Fh5g42590* encodes TATA-box binding protein associated factor (TAF), which plays a role in seed development. *Fh5g09460* encodes a growth-regulating factor (GRF), which plays a role in leaf development. *Fh7g14670* encodes purple acid phosphatase (PAP), which regulates cell wall synthesis. *Fh6g11380* encodes the aldo keto reductase superfamily (AKR4C), which is involved in the photosynthesis process. *Fh4g19990* encodes HtrA 1 (HTA1), which responds to various adverse stimuli. In summary, we screened 15 candidate genes related to citrus leaf size through RNA-seq analysis. The results provide a theoretical basis for a deeper understanding of the molecular mechanism of citrus leaf size and offer new genetic resources for the study of citrus leaf size.

### 3.6. qRT-PCR

**Figure 8:**
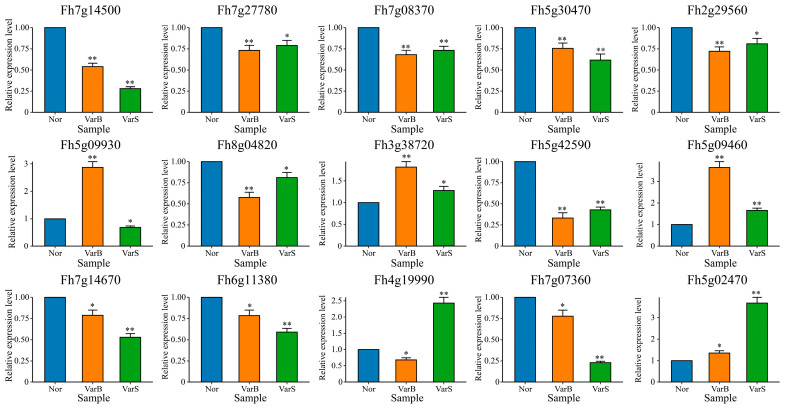
Analysis of hub gene expression patterns in different materials. (Error bars represent the mean ± SE of three replicates, * *p* < 0.05; ** *p* < 0.01).

The expression patterns of the 15 hub genes in different materials were detected via qRT-PCR (Figure 8). The expression levels of 10 genes (*Fh7g14500*, *Fh7g27780*, *Fh7g08370*, *Fh5g30470*, *Fh2g29560*, *Fh8g04820*, *Fh5g42590*, *Fh7g14670*, *Fh6g11380*, and *Fh7g07360*) in VarB and VarS were significantly lower than those in Nor. Among them, the expression levels of five genes (*Fh7g14500*, *Fh5g30470*, *Fh7g14670*, *Fh6g11380*, and *Fh7g07360*) in VarS were the lowest. Three genes (*Fh5g09930*, *Fh3g38720*, and *Fh5g09460*) were most highly expressed in VarB, and two genes (*Fh4g19990* and *Fh5g02470*) were most highly expressed in VarS.

## 4. Discussion

Leaves are vital for photosynthesis, serving as the primary location for this process and ranking among the essential photosynthetic organs in plants. The size of leaves directly affects the area of light received by the plant and thus has a direct effect on crop yield [35]. The yield of crops is generally affected by two factors, namely, the potential of the “source” and the size of the “sink” [36]. The main factor affecting the potential of the “source” is photosynthetic efficiency, whereas the main factors affecting the size of the “sink” include the shape of the panicle and the size of the grain or fruit [37]. Leaves are among the most important photosynthetic organs of plants and the most important “source” in the growth process of plants. Studies have shown that approximately 95% of the dry matter in crop production comes from photosynthetic products [38]. Flag leaves, second leaves and third leaves play vital roles in the yield of grass crops [39]. Rice flag leaf photosynthesis provides up to 60% of the carbohydrates needed for endosperm formation. Studies on rapeseed have shown that adequate potassium nutrition can increase the photosynthetic rate by increasing the leaf area, thereby increasing crop yield [40]. *CsRAXs* regulate the content of free auxin in leaves through *CsUGT74E2*-mediated auxin glycosylation to regulate leaf size and stem thickness, thereby affecting cucumber yield [41]. In this study, transcriptome sequencing of leaves from ‘Cuimi’ kumquat (Nor) and two grafted materials with larger leaves (VarB) and smaller leaves (VarS) was performed. A total of 7264 DEGs were identified in the three materials, including 2374 transcription factors, and 254 genes were differentially expressed among the three materials. The DEGs were enriched in pathways related to sugar metabolism, cell wall composition, starch biosynthesis, and photosynthesis. Weighted gene coexpression network analysis revealed 15 candidate genes associated with citrus leaf size, including three encoded transcription factors: *Fh5g30470* (MYB), *Fh7g07360* (AP2/ERF), and *Fh5g02470* (SAP). Relative to Nor, *Fh7g14500* (*FhABC*) and *Fh5g42590* (*FhTAF*) were downregulated in both VarB and VarS. *Fh7g08370* (*FhGORAB*), *Fh7g27780* (*FhTBL*), *Fh2g29560* (*FhABC*), *Fh7g14670* (*FhPAPs*), *Fh6g11380* (*FhAKR4C*), and *Fh7g07360* (*FhAP2/ERF*) were mildly downregulated in VarB and VarS. *Fh5g30470* (*FhMYB*) was downregulated in VarB and VarS, but its expression level in VarS was lower than that in VarB. *Fh5g09930* (*FhRRP*) is mildly downregulated in VarS but upregulated in VarB. *Fh3g38720* (*FhAHL10*) and *Fh5g09460* (*FhGRF*) were upregulated in VarB and VarS, but their expression levels in VarB were greater than those in VarS. *Fh4g19990* (*FhHTA1*) is mildly downregulated in VarB but upregulated in VarS. *Fh5g02470* (*FhSAP*) was upregulated in VarB and VarS, but its expression level in VarS was greater than that in VarB. *Fh5g09930* (*FhRRP*) had the highest expression level in VarB, the middle expression level in Nor, and the lowest expression level in VarS, indicating that *Fh5g09930* (*FhRRP*) may be a positive regulator of citrus leaf size. *Fh4g19990* (*FhHTA1*) had the lowest expression level in VarB, the middle expression level in Nor, and the highest expression level in VarS, indicating that *Fh4g19990* (*FhHTA1*) may be a negative regulator of citrus leaf size. Although *Fh5g09460* (*FhGRF*) and *Fh7g07360* (*FhAP2/ERF*) presented the highest or lowest expression levels in Nor, their expression levels were consistent with the phenotypes of different leaf sizes, and these genes could also be used as important candidate genes for the positive regulation of citrus leaf size. Further evaluations of these findings are undoubtedly the goal of future functional genomics research.

Sugar metabolism is an important biochemical process in plant leaves and plays an important role in plant leaf development, providing energy and carbon for construction, growth and the maintenance of physiological metabolism [42]. Sugars promote cell wall synthesis and influence cell wall plasticity and relaxation, impacting leaf expansion and growth [43]. Moreover, sugars can also regulate the plasticity and relaxation of the cell wall, affecting the expansion and growth of leaves. Sugar metabolism plays an important role in chloroplast development and chlorophyll synthesis [44]. Chloroplasts are the main site of photosynthesis, and they need to synthesize and accumulate enough chlorophyll to effectively absorb light energy. Sugars, as the main source of glucose and other sugars, provide energy and carbon sources and affect the formation and function of chloroplasts [45]. Photosynthesis, which converts light energy into chemical energy and synthesizes sugars and other organic substances, is an important process that occurs in leaves. Sugar metabolism regulates the distribution of photosynthetic products to different target organs, such as leaves, stems, and roots [46]. This distribution determines the direction and process of plant growth. Through enrichment analysis of the 2636 DEGs between VarS and VarB revealed in this study, we also identified pathways such as sugar metabolism, cell wall composition, starch biosynthesis, glycolysis/gluconeogenesis, starch and sucrose metabolism, the pentose phosphate pathway, and photosynthesis pathways. Eight of the fifteen candidate genes (*Fh2g29560* (*FhABC*), *Fh3g38720* (*FhAHL10*), *Fh5g02470* (*FhSAP*), *Fh5g09460* (*FhGRF*), *Fh6g11380* (*FhAKR4C*), *Fh7g14500* (*FhABC*), *Fh7g14670* (*FhPAPs*), and *Fh7g27780* (*FhTBL*)) were significantly enriched in these pathways.

The cell cycle was also significantly enriched in the enrichment analysis. Studies have shown that during leaf development, the regulation of the cell cycle also plays an important role in the proliferation and expansion of leaves [47]. During the formation and growth of leaves, the number of cells must increase through cell division [48]. The G1 phase of the cell cycle is a key stage for cell proliferation [49]. During the G1 phase, cells obtain sufficient nutrients and growth conditions to prepare for subsequent cell division. Cells also need to expand by increasing their volume. The G2 phase of the cell cycle is a key stage for cell expansion, as it is the transition between cell enlargement and cell proliferation [50]. During the G2 phase, cells continue to synthesize proteins and accumulate sufficient organelles and cytoplasm to provide a sufficient material basis for cell division [51]. The control of the cell cycle determines when cells stop proliferating and begin to differentiate [52]. Some studies have shown that changes in the expression profiles of cell cycle proteins and cell cycle regulatory genes during leaf development are closely related to cell differentiation [53]. The cell cycle is also closely related to leaf development. Different stages of the cell cycle provide leaves with the regulatory mechanisms required for cell proliferation, expansion and differentiation. The study of DEGs during the cell cycle helps us better understand the molecular mechanism of citrus leaf development. Notably, we also discovered that *Fh5g30470* (*FhMYB*) and *Fh8g04820* (*FhATM*) are candidate genes that are involved in regulating plant growth and development and the cell cycle.

The AP2/ERF family of genes comprises unique transcription factors found in plants. These genes play key roles in plant growth, hormone-induced development, the ethylene response, and the stress response [54]. Some members of this family also play important roles in leaf development and can regulate leaf cell proliferation and expansion, thereby affecting leaf size. AP2/ERF transcription factors can indirectly affect leaf size by regulating the expression of cell cycle proteins, affecting the cell division rate and cell proliferation [55]. They can also affect the regulation of cell wall biosynthesis and metabolic pathways, thereby indirectly affecting the expansion process of cells and influencing leaf size [54]. Light signals are important regulatory factors in plant leaf development, and some AP2/ERF transcription factors can act as key molecules that mediate light signal transduction and affect leaf size. In addition, some members of the AP2/ERF family can also regulate the expression of other genes related to leaf size, such as those involved in the regulation of key processes such as chloroplast development and chlorophyll synthesis, thereby affecting leaf size. In *Liriodendron chinense*, three AP2/ERF transcription factors (*LcERF94*, *LcERF96*, and *LcERF98*) are associated with early leaf development and morphogenesis, with high expression levels in both the SAM and leaf primordia [56]. In *A. thaliana* and tobacco, overexpression of the AP2/ERF transcription factor *BOLITA* (BOL) leads to reductions in cell size and number, resulting in smaller leaves [57]. In addition, *SsAP2/ERFs* are widely expressed in the leaves of mature sugarcane, indicating that they play important roles in the growth and development of sugarcane [58]. We identified a candidate gene, *Fh7g07360* (*FhAP2/ERF*), encoding an AP2/ERF transcription factor whose homologous gene in *A. thaliana* is involved in regulating the development of cotyledons and leaves. In summary, the pathways and candidate genes we identified can serve as the focus of citrus leaf development research and provide important information for subsequent studies.

## 5. Conclusions

In this study, we performed transcriptome sequencing on the leaves of ‘Cuimi’ kumquat (Nor) and two leaf variants, namely, one with larger leaves (VarB) and one with smaller leaves (VarS). We defined several important regulatory pathways involved in citrus leaf development by identifying DEGs and differentially regulated transcription factors among the materials. In addition, we screened 15 candidate genes related to citrus leaf development through WGCNA, which included three transcription factors. However, the exact roles of these genes in citrus leaf development remain to be determined. Our results provide a theoretical basis for a deeper understanding of the molecular mechanism of citrus leaf development and offer new genetic resources for citrus leaf research.

## Figures and Tables

**Table 1:** Functional annotation and sources of candidate genes related to citrus leaf size.

Gene id	*Arabidopsis thaliana* Homologous Gene	Gene Name	Gene Annotation	Source	Leaf Phenotype
*Fh7g14500*	AT3G48770	ATP-binding cassette transporter proteins (ABC)	Regulates growth and development	VarB	larger leaves
*Fh7g27780*	AT1G60790	Trichome birefringence-like (TBL)	Synthesis and deposition of secondary wall cellulose	VarB	larger leaves
*Fh7g08370*	AT3G52900	RAB6-interacting golgin (GORAB)	Microtubules required for immunity	VarB	larger leaves
*Fh5g30470*	AT1G01520	Transcription Factor (MYB)	Regulates circadian rhythms	VarB	larger leaves
*Fh2g29560*	AT1G30410	ATP-binding cassette (ABC)	Regulates growth and development	VarB	larger leaves
*Fh5g09930*	AT1G12650	rRNA biogenesis RRP-like protein (RRP)	Processing and capping of rRNA in the nucleus and cytoplasm	Nor	Normal leaves
*Fh8g04820*	AT2G15570	Arabidopsis thioredoxin M-type (ATM)	Involved in activating the cell cycle and DNA damage repair	Nor	Normal leaves
*Fh3g38720*	AT2G33620	AT-Hook-Like10 (AHL10)	Can inhibit the maturation of shoot apical meristems	Nor	Normal leaves
*Fh5g42590*	AT1G02680	TATA-box binding protein associated factor (TAF)	Plays a role in seed development	Nor	Normal leaves
*Fh5g09460*	AT2G36400	Growth-regulating factor (GRF)	Plays a role in leaf development	Nor	Normal leaves
*Fh7g14670*	AT5G23690	Purple acid phosphatase (PAPs)	Regulates cell wall synthesis	VarS	smaller leaves
*Fh6g11380*	AT2G37770	Aldo keto reductase superfamily (AKR4C)	Participates in photosynthesis	VarS	smaller leaves
*Fh4g19990*	AT5G54640	HtrA 1 (HTA1)	Response to various adverse stimuli	VarS	smaller leaves
*Fh7g07360*	AT1G12980	Transcription Factor (AP2/ERF)	Involved in regulating embryogenesis	VarS	smaller leaves
*Fh5g02470*	AT5G35770	Stress-Associated Protein (SAP)	Regulates inflorescence, flower and ovule development	VarS	smaller leaves

## Data Availability

The RNA-seq data presented in the study are deposited in the NCBI repository under accession number PRJNA1172372.

## Supplementary Materials

The following supporting information can be downloaded at https://www.mdpi.com/article/10.3390/genes16010097/s1: Table S1. Information on all primers used in this study; Table S2. Transcriptome data quality statistics of the test samples. Table S3. Gene coexpression network within the green, turquoise, and yellow modules.
